# Solvent Models and Charge Scaling: Benchmarks for Molecular Dynamics of Glycosaminoglycans

**DOI:** 10.1021/acs.jpcb.6c01164

**Published:** 2026-04-29

**Authors:** Jacob A. Clark, Sergey A. Samsonov

**Affiliations:** Faculty of Chemistry, 49646 University of Gdansk, Wita Stwosza 63, Gdansk 80-308, Poland

## Abstract

Glycosaminoglycans make up a group of highly negatively charged linear polysaccharides with a wide variety of physiological roles. Investigating these biomolecules requires both experimental and computational approaches. However, there is limited understanding of how various parameter choices in the design of simulations can impact the behavior of glycosaminoglycans. Previous work within our group has explored the impact of solvent model choice on unbound glycosaminoglycans and within their complexes with proteins, finding dramatic differences in results that depend on which solvent model is used. The high negative charge these molecules possess also poses a challenge, as the simulations become not only dependent on solvent model choice but also sensitive to changes in forcefield parameters. Charge scaling methods have been proposed to improve the accuracy of forcefields used to simulate glycosaminoglycans. In this study, the application of charge scaling methods within the context of the solvent model has been rigorously investigated with the goal of quantifying the impacts of distinct protocols in the analysis of protein–glycosaminoglycan interactions. Utilizing previous experimental data collected on glycosaminoglycan chain length for reference, it was found that charge-scaled simulations of heparin resulted in greater similarity to experimental properties than conventional non-charge-scaled approaches. This improvement in the simulated properties of heparin is maintained with multiple solvent models and under typical scenarios in which heparin may be simulated, along with characterization of the effects on protein–glycosaminoglycan binding, provides a more comprehensive understanding of how the challenge of solvent model choice and forcefield sensitivity can be ameliorated within the field of glycosaminoglycan simulations.

## Graphical abstract

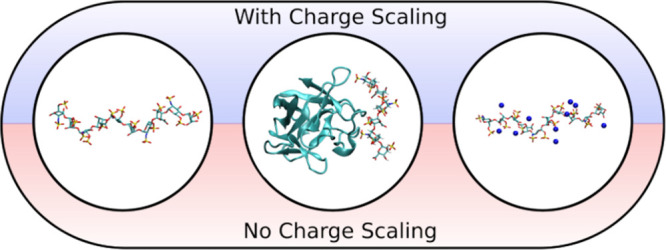

## Introduction

Glycosaminoglycans (GAGs) are long-chain negatively charged polysaccharides that are, with few exceptions,^1^ linear structures. They consist of repeating disaccharide units of a hexuronic acid (glucuronic acid or iduronic acid) and a hexosamine (galactosamine or *N*-acetylate glucosamine),^2^ with the exception of keratan sulfate (galactose in place of hexuronic acid). The interchangeable nature of the disaccharide repeating unit, along with the capacity for sulfation throughout these saccharides at various positions, results in a very high degree of structural heterogeneity and by extension a broad array of functionality.^3^ GAGs are expressed extensively at the cell surface and within the extracellular matrix and are key components of proteoglycans (PGs). The structure of the GAGs involved, influences the biological activity of the PG.^4^ GAGs are present across the body, located within blood vessel walls, lung tissue, intestinal mucosa, the liver, kidneys, and nervous tissue.^5^ Due to their presence within so many tissues and vital organs, the importance of these molecules in the field of glycobiology cannot be overstated. GAGs have multiple roles, both in a structural capacity within the extracellular matrix supporting and connecting cells within tissues, and also in cell signaling inducing effects via regulating protein activity, such as in growth factors and chemokines.^6^ Evidence of GAG activity has been demonstrated for processes, such as ligand-binding, regulation of cellular activity, homeostasis, and regulation of enzyme behavior.^3^ The action of GAGs within these systems is key to understanding various pathologies, including cardiovascular disease,^7^ cancer,^8^ neurodegenerative diseases,^9^ as well as inflammation^10^ and healing mechanisms.^11^

Understanding the role GAGs play within cellular processes and the machinery involved at an atomic level requires both extensive experimental exploration and computational modeling. While experimental work has outlined physiological roles and specific targets GAGs interface and mediate effects through,^3^ the mechanism by which these molecules act is still not fully understood. Molecular modeling offers an approach to understand GAG functionality at a high temporal and spatial resolution; however, due to the high degree of structural heterogeneity and high charge density, there is substantial practical difficulty in developing accurate models.^3,12^ Structural heterogeneity makes the volume of possible structures and the identification of specific relevant options difficult, and the high charge density makes the simulation results very dependent on the forcefield used. Computational modeling has been carried out both at the coarse-grained^13,14^ and atomistic^15^ levels; however, key considerations have been identified as obstacles to improved GAG simulations. Two major hurdles addressed in this work are the impact of the solvent model choice and the high charge density of GAGs. The question of how solvent models impact these simulations is critical to answer for two reasons, first, due to the important role of water in protein-GAG systems being demonstrated^16^ and, second, the ubiquitous use of TIP3P as a standard for molecular dynamics (MD), evidenced by over 37,000 citations of the original model.^17^ It has been established that depending on the choice of solvent model, GAGs will demonstrate different structural and dynamic properties.^18,19^ Several studies have experimented with multiple solvent models, increasing in complexity from the standard three-point explicit models (TIP3P, OPC, and SPC) up to the computationally demanding four- and five-point models (TIP4P and TIP5P). Previous work in our group has illustrated the drawbacks of the TIP3P model specifically through comparison of the effects of TIP3P, OPC, SPC/E, TIP4P, and TIP5P models, as well as the less demanding implicit models on GAG behavior,^19,20^ using the model GAG, heparin (HP). Calculation of general descriptors of the GAG molecules, such as end-to-end distance (EED), hydrogen bonding, glycosidic linkage populations, and radius of gyration showed that TIP3P in this work led to a less accurate model of the GAG simulated compared to the more complex solvent models like TIP5P. The folding nature of GAGs in simulations has been documented previously, where the structure forms a “U”-shape.^19,21^ This was reproduced in our group when comparing simulations using various solvent models, highlighting the issue with TIP3P in comparison to more complex solvent models like TIP5P, where simulations showed a more extended molecule in line with expected HP behavior.^22^ This work was built upon to further probe the impact of solvent models on GAGs within the context of protein binding, implementing the same approach in a system of HP bound with fibroblast growth factor (FGF). Applying the same methods to describe the systems, it was found that solvent model choice again altered the behavior of the system, leading to an overestimation of binding energies with most used models, with simulations using the implicit models demonstrating the worst exacerbation of this issue.^20^

Beyond the choice of solvent model, the role of cations has been experimentally verified and their critical role in protein–GAG interactions has been demonstrated repeatedly.^23–25^ In particular, the role of calcium ions has been highlighted both experimentally and with detailed computational modeling and so to accurately model GAGs, their presence in a system needs to be taken into account.^26,27^

Along with solvent model choice and consideration of cation interactions, the forcefield itself plays a big role in determining GAG behavior, more so than most systems due to the high negative charge of GAGs. Conventional forcefield approaches applied to GAGs result in overestimated electrostatic interactions and subsequent issues of unreliable binding energies and artifact dynamic behavior.^28^ Utilizing higher complexity polarizable forcefields can mitigate this issue and improve GAG behavior;^29^ however, these types of forcefields are not available for sulfated GAGs and are frequently computationally demanding. An alternative approach that has been developed is charge scaling, a method of processing the partial charges of a molecule to recapture the missing electronic part of the dielectric constant of water into Coulomb’s law, emulating the polarizing effect of a solvent without the computational demand of a fully polarizable forcefield. The electronic continuum correction (ECC), as this approach is called, has been demonstrated to improve electrostatic interactions in electrolytes,^30^ phosphates,^31^ and proteins, such as ion channels,^32^ membranes,^33^ and relevant to the focus of this work, GAGs.^28^ The work presented here builds upon prior solvent model simulations by implementing this charge scaling technique.

By using previous benchmark simulations as a control for charge-scaled systems for each solvent model, a more comprehensive understanding can be gained of what the impacts of both charge scaling and solvent model choice have on GAGs behavior and highlights the utility of these methods for both more reliable and more efficient simulations of GAGs. While this prior work has investigated the impact of solvent model alone,^19^ or the performance of charge scaling with regard to specific cases,^28^ a comparative analysis of the charge scaling method for results with solvent models against experimental data has not been carried out. In the work presented here, a series of simulations are shown in which the implementation and effect of charge scaling on GAGs is investigated, within the context of solvent model used and protein-GAG and GAG–cation interactions. The data collected is intended to aid in building a framework for understanding how choices made in building a system can influence the behavior of these molecules and inform future endeavors to be more accurate in modeling the behavior and dynamics of GAGs.

## Methods

### Structures and Parameterization

The starting structure of the HP was the dp10 (dp stands for degree of polymerization) form obtained from the protein data bank (PDB),^34^ PDB ID: 1HPN.^35^ The sulfate group charges were taken from literature data^36^ and the GLYCAM06 forcefield parameters were used.^37^ To ensure the data produced previously with this system was appropriate to be used as a control against simulations with charge scaling implemented, the IdoA2S in ^1^C_4_ conformation, which is dominant in HP,^38^ present in these particular crystal structures was used as a starting conformation in the simulations without any restraints applied for the puckering in the MD run. For the protein systems, the protein–GAG complex used was taken from the PDB, with the bound HP parametrized in the same way (PDB ID: 1BFC). For the protein, the ff14SB forcefield was used,^39^ and the default AMBER parameters from AMBER16 ^40^ were used for the calcium ions, where they are treated as spheres, and their interactions are exclusively determined by Lennard-Jones parameters and a charge localized in a single point.

### Water Models

The parameters for the water models were all taken from AMBER16,^40^ and the recommended mbondi parameters were applied for each particular model. The same comprehensive list of water models was used in the prior research,^19,20^ both in the HP and FGF-HP system with implicit models: IGB 1, 2, 5, 7, and 8 and explicit models: TIP3P, SPC/E, OPC, TIP4P, TIP4PEw, and TIP5P.

### Molecular Dynamics Simulations

The MD simulations were carried out with the AMBER package.^41^ For the free HP simulations, the decasaccharide was solvated in an octahedral periodic box with a solute to box minimum distance set to 8.0 Å and neutralized with counterions (Na^+^ for the free HP and Cl^–^ in the FGF-HP complex simulations) in the explicit solvent simulations; no counterions were required for the implicit solvent simulations, as is standard practice in AMBER. The no “saltcon” option was implemented for the implicit solvent simulations. Energy minimization consisted of two steps, the first 1.5 × 10^3^ steepest descent cycles and 10^3^ conjugate gradient cycles, while harmonic force restraints were in place on solute atoms. The second step involved 10^3^ steepest descent cycles and 3 × 10^3^ conjugate gradient cycles without restraints in the case of explicit simulations. The heating procedure involved heating to 300 K for 10 ps, while harmonic force restraints of 100 kcal mol^–1^ Å^–2^ were in place on solute atoms. Equilibration was carried out for 50 ps at 300 K and 10^5^ Pa in the isothermal isobaric ensemble (*NPT)* in the case of explicit solvent simulations with the Berendsen barostat and Langevin thermostat implemented with default AMBER parameters. Production was then carried out in the *NPT* ensemble also for explicit solvent systems, and for all systems, the SHAKE algorithm, a 2 fs time step, 8 Å cutoff for nonbonded interactions, and the Particle Mesh Ewald method were applied, and run for 10 microseconds. The protein–GAG systems were set up similarly, the key difference for the protein–GAG system being the use of a 12 Å solute–box border distance and the use of the ff14SB forcefield parameters for the protein. The simulations carried out to investigate the calcium ion interactions were designed with the same parameters as the free HP simulation, with the addition of 10 calcium ions.

### Charge Scaling

The protocols detailed in Riopedre-Fernandez et al.^28^ were followed to incorporate electronic polarization in a mean-field way. The specific method involved the scaling down of the partial charges of carboxyl and sulfate groups uniformly by a factor of 0.75. The procedure itself followed exactly the protocols laid out in the literature and was applied to equivalent groups to produce the same GLYCAM-ECC75. The ECC protocol accounts for electronic polarization by modeling charged moieties in water with a dielectric constant of water ϵ_e*l*_ = 1.78, corresponding to a refractive index of $n=\sqrt{\epsilon_{\mathrm{el}}}=1.3432$ .^32^ This leads to a charge scaling factor of $1/\sqrt{\epsilon_{\mathrm{el}}}=0.75$ , which is appropriate for biological systems. After scaling the charges, integer total charges are restored by manually adjusting the partial charges of the carbon atoms to which the charged groups are attached, ensuring that other internal molecular interactions are not affected. The adjusted charges for the whole ionic group of HP were then integrated into GLYCAM libraries used for the simulation. Implementation of the ECC approach with the GLYCAM model like this is referred to as the GLYCAM-ECC75 variant.^28^

### Trajectory Analysis

A series of MD-derived general descriptors were used to analyze the trajectories and the behavior of the HP molecule. This included radius of gyration, EED, hydrogen bonding, ring puckering, and dihedral angle analysis, all analyzed using cpptraj AMBERTools scripts. Contact analysis for the protein–GAG system was also carried out in cpptraj with a 7 Å interatom distance cutoff. In contact analysis, the native contacts can be defined as those that are within the cutoff in the reference structure used, while non-native contacts are contacts within the cutoff that are not in the original reference.

## Results and Discussion

### Heparin

To observe the properties of HP and discern changes resulting from implementation of the ECC, HP was simulated for 10 μs with each solvent model. The data was compared to the previous works on simulating HP with solvent models, as given in Marcisz and Samsonov 2023.^19^

Similar to the previous work, the biggest differences observed were between HP simulated with explicit and implicit models. The general descriptors calculated for each simulation and their respective solvent models used are illustrated in Tables 1 and 2. The EEDs provide information on how elongated the structures are. This calculation measures the distance between the terminal atoms at either end of the molecule and so any compression or extension of the molecule is detectable, correlating with the bending of the molecule. The EEDs shown in Table 1 demonstrate how the HP in simulations with implicit solvent are in a more extended state than those in explicit solvent; this extension is beyond experimental values previously reported.^22,42^ The shape of HP in the structural ensembles obtained in MD is illustrated in Figure 1. The plausible explanation for the HP increased length in simulations using the implicit models is the lack of counterions present in the solvent, which are frequently found in proximity to HP and likely led to more pronounced curvature of HP via counterion–sulfate interactions.^19^ Of the simulations with explicit water models, most are similar to the exception of those using TIP4PEw, which deviates to a degree smaller by around 1 Å.

**Table 1:** General Descriptors in Simulations across Explicit Solvent Models^a^

	TIP3P	TIP4P	TIP4P-Ew	TIP5P	OPC	SPC/E
EED (Å)	27.2 ± 4.6	27.0 ± 4.6	25.9 ± 5.0	27.5 ± 4.6	26.8 ± 4.7	27.0 ± 4.6
Fluct (Å)	2.9 ± 0.7	3.3 ± 0.9	3.9 ± 1.3	3.1 ± 0.8	2.8 ± 0.7	3.0 ± 0.8
RMSD (Å)	3.3 ± 0.9	3.5 ± 1.2	3.8 ± 1.8	3.7 ± 1.1	3.7 ± 0.9	3.0 ± 1.0
Radgyr (Å)	12.7 ± 0.5	12.5 ± 0.7	12.3 ± 1.1	12.7 ± 0.5	12.7 ± 0.5	12.7 ± 0.6

a Experimentally derived EED = 27.6 Å.

**Table 2:** General Descriptors in Simulations across Implicit Solvent Models

	IGB1	IGB2	IGB5	IGB7	IGB8
EED (Å)	38.7 ± 3.4	39.0 ± 2.9	37.9 ± 3.2	40.0 ± 2.6	40.8 ± 3.3
Fluct (Å)	3.6 ± 0.7	3.7 ± 0.7	3.7 ± 0.8	3.3 ± 0.7	3.5 ± 0.7
RMSD (Å)	4.2 ± 1.1	4.8 ± 0.9	4.2 ± 0.9	4.0 ± 0.8	4.1 ± 0.9
Radgyr (Å)	12.8 ± 0.6	12.8 ± 0.5	12.6 ± 0.6	13.0 ± 0.5	13.4 ± 0.7

**Figure 1:**
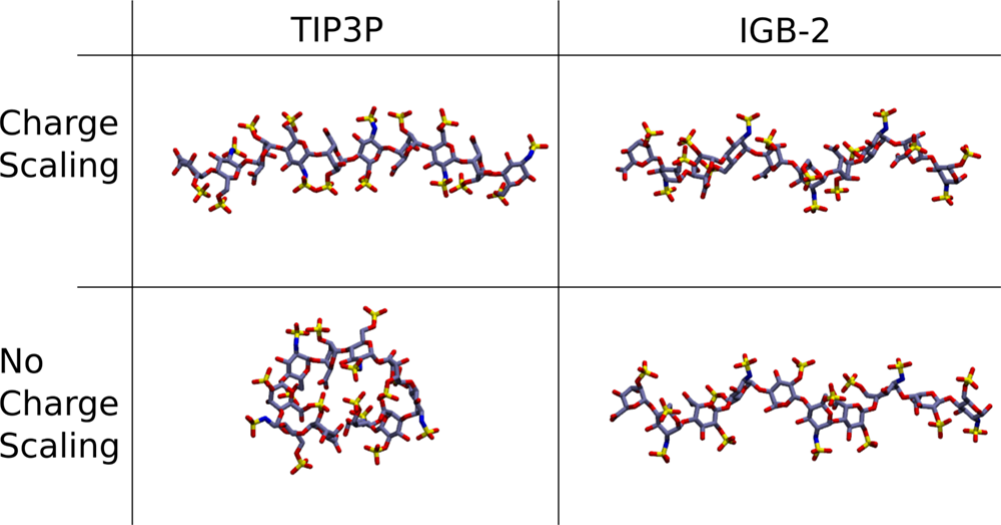
Shape of HP in the simulations with an explicit solvent and implicit solvent, using TIP3P and IGB-2 as examples. The charge-scaled results are shown alongside results from the previous work without charge scaling.^19^

To compare the EEDs obtained in the simulation of HP dp10 with the experimental values (27.6 Å for dp10 ^22^), we normalized the data per dp, since the data from the experiments corresponded to HP of dp8, 12, 20, 24, and 36. To do this, we took the EEDs derived from SAXS experiments and divided the values by the dp of the molecule to get an average that could be scaled for the dp10 system used here. This puts the explicit solvent simulation values close to the expected length, whereas the simulations with implicit solvents overshoot this EED, by over 13 Å in the case of IGB8. The radii of gyration values were similar across the explicit and implicit model simulations, with a minor increase in the implicit group. All values were close to experimental results that have been found to be 12.8 Å,^35^ the explicit solvent simulations other than TI4PEw and the implicit model simulations IGB1 and IGB2 being the closest. The root mean square deviation (RMSD) values were calculated from the starting frame of the production simulation for the heavy atoms, and the higher RMSD values in the implicit solvent simulations reflect that the structures obtained with this method are more extended in comparison to the experimental structure (Figure 1). The atomic fluctuations mirror the results of the RMSD with higher values obtained when implicit solvents were used compared to use of explicit models, with exception to the simulation using TIP4PEw, which had the highest result. The exceptional values for TIP4PEw come from rare behaviors within the trajectory, where the HP folds over and interacts with itself, in a similar way to the non-charge-scaled systems; these events can be seen in the Supporting Information RMSD time-series Figure S1.

These descriptors were then compared to the one from the previous work carried out without the charge scaling protocol being implemented to discern changes brought about by the altered charges. The resulting differences are highlighted for the EEDs in the violin plot in Figure 2. Here, the distributions of the EEDs over the course of the simulations can be compared. It can be observed that the charge-scaled data are substantially shifted higher in simulations using explicit solvent, bringing the values and subsequent level of extension of the molecules closer to the experimental value of 27.6 Å. This shift is more pronounced in simulations with lower EEDs without charge scaling applied, such as TIP3P, TIP4P, TIP4PEw, and SPC. Less of a drastic shift in EED is seen in the simulations using the TIP5P and the OPC solvent models. In the case of the implicit solvent use, the shift in EED is inverted, with lower values also closer to expected values but still correspondning to more extended structures. To demonstrate the stability of the obtained conformational ensembles, the time-series results for the EEDs over the course of the production can be seen in Figure S2.

**Figure 2:**
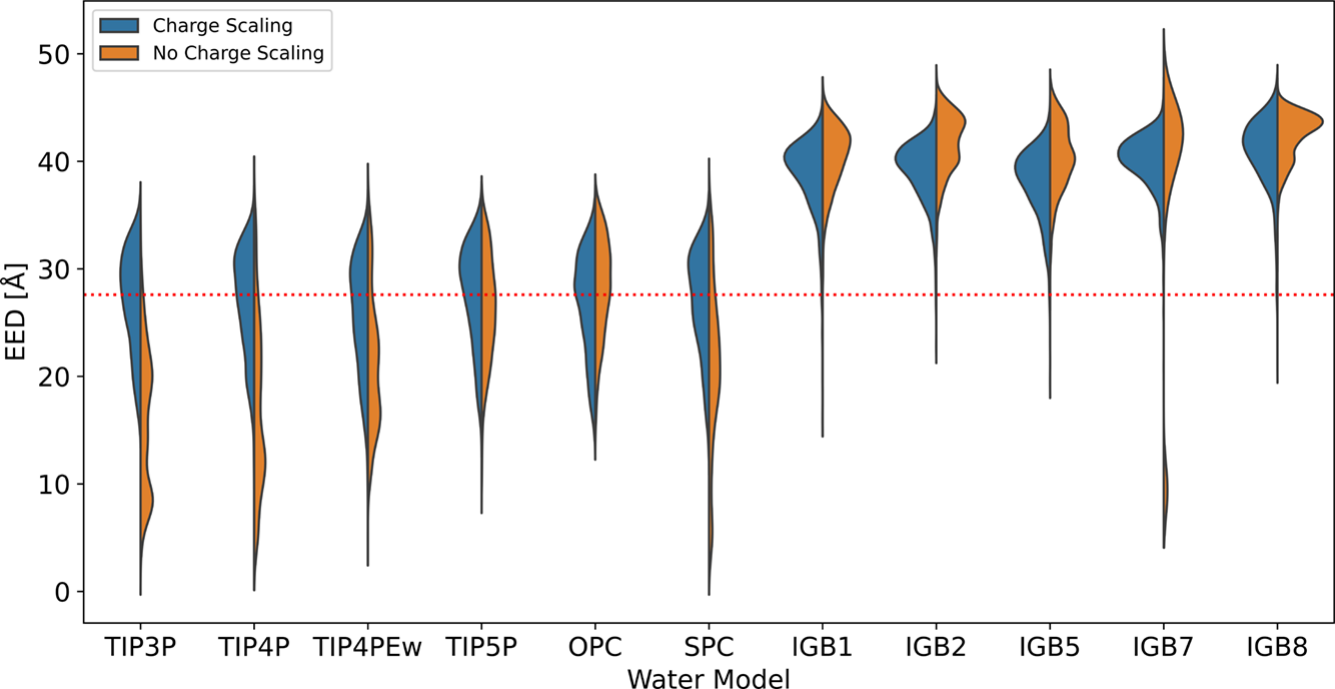
Distributions of the EEDs plotted for each simulation with solvent model choice labeled. The non-charge-scaled data was taken from Marcisz and Samsonov 2023 ^19^ and plotted in orange. The red dotted line indicates the experimental EED value.

Intramolecular hydrogen bonding was also analyzed to evaluate how HP was folding and interacting with itself. Most hydrogen bonds were formed between atoms on the same residue, similar to prior work without charge scaling; however, the big difference was in the quantity of hydrogen bonds. There is a reduction in the number of hydrogen bonds in the case of the charge-scaled simulations for almost all solvent models used, illustrated in Figure 3. The hydrogen bonds involved in maintaining the “U”-shaped conformations reported in simulations without charge scaling are not present anymore, aligning with the EED results that demonstrate more extended structures. Prior work cited clustering of counterions to bridge negatively charged HP groups to enable the folded conformation to form.^19^ The charge scaling in these simulations reduces the potency of these interactions and prevents this clustering and folding effect to produce an extended structure with fewer internal hydrogen bonds and an EED matching experimental values. A time-series plot of these internal hydrogen bonds can be seen in Figure S3, providing clarity on the persistence of these hydrogen bonds.

**Figure 3:**
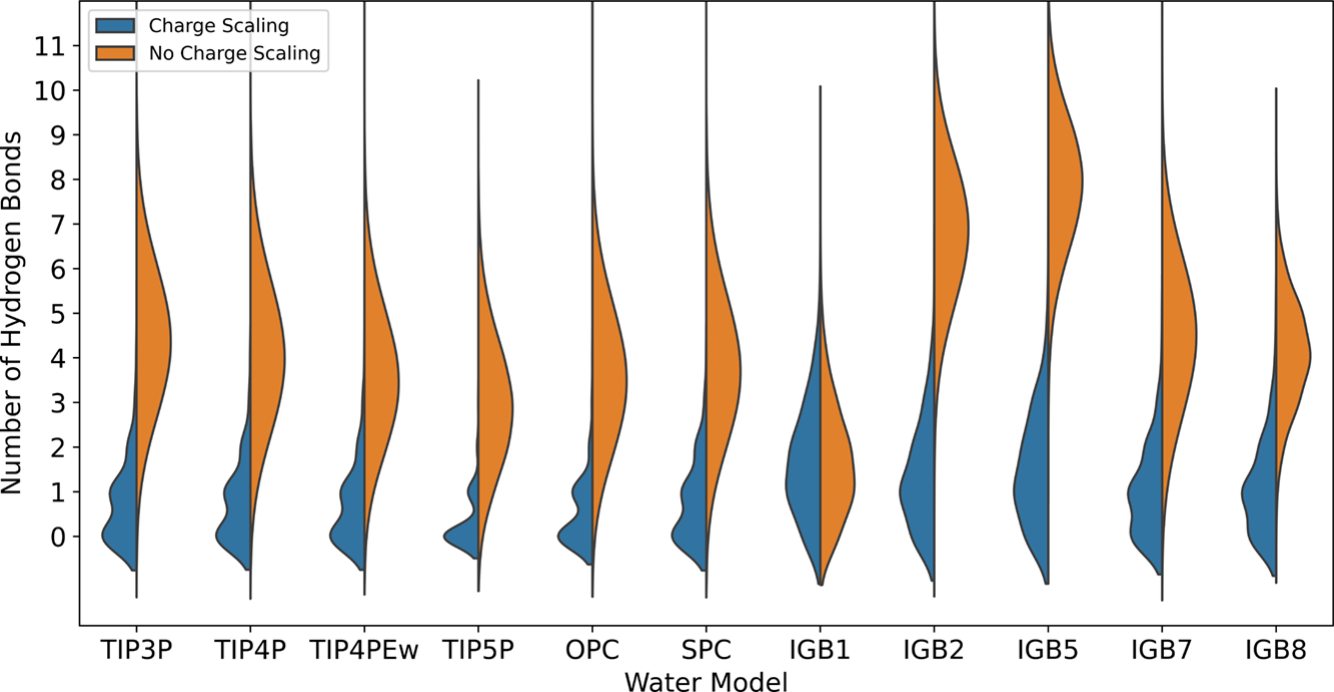
Distribution of the number of internal hydrogen bonds calculated for each simulation, the non-charge-scaled data taken and plotted from Marcisz and Samsonov 2023.^19^

Furthermore, dihedral angles across the glycosidic linkages were analyzed to get the populations of both GlcNS6S–IdoA2S and IdoA2S-GlcNS6S angles. For simulations with TIP3P (Figure 4), we see that the GlcN6S-IdoA2S dihedral is mostly found to be around ϕ – 80° and ψ 125°. For the IdoA2S–GlcN6S dihedral, the angle is mostly ϕ 80° and ψ 100°. These results match experimental data^38^ but differ slightly with prior computational work without charge scaling,^19^ where an additional conformation in GlcNS6S–IdoA2S was found at ϕ −80° and ψ −50°. This conformation, however, is linked to the curvature of HP and, therefore, its absence is expected given the more extended structure as a consequence of charge scaling implementation. The results are consistent across the simulations with explicit solvent, but the systems using implicit solvent still demonstrate this second conformation to some degree. The complete data across explicit solvent simulations for the glycosidic linkage dihedral results can be found in Figure S4.

**Figure 4:**
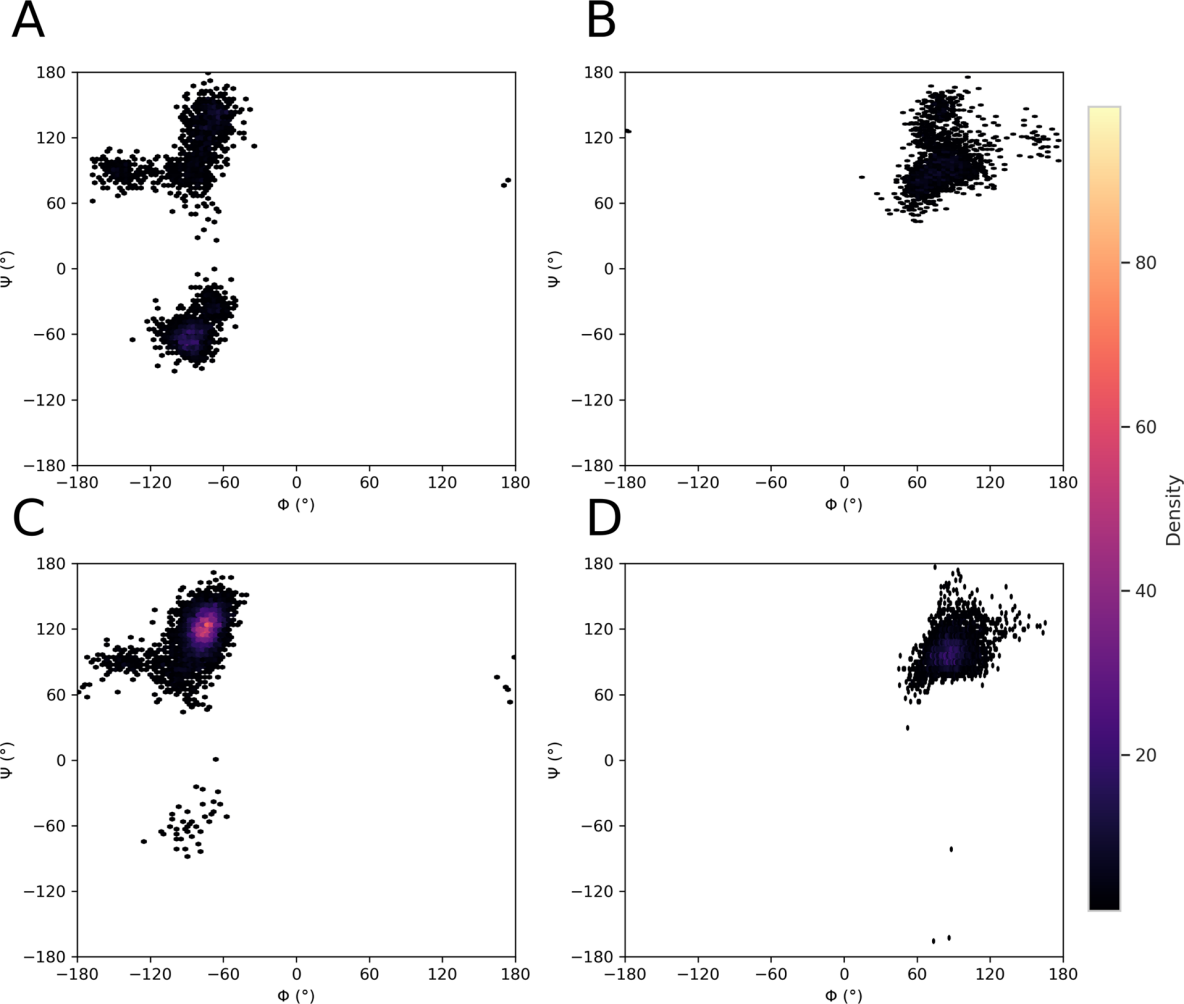
Distribution of dihedral angles measured from the TIP3P water model simulation. GlcNS6S–IdoA2S dihedrals are shown on the left without and with charge scaling, (A) and (C), respectively. The IdoA2S–GlcNS6S dihedrals without and with charge scaling are shown on the right, (B) and (D), respectively.

### FGF-HP Complex

Furthermore, to understand the processes through which GAGs act on proteins to mediate their biological function, reliable computational approaches are needed. Here, we present results from MD simulations of an FGF-HP complex utilizing the same methodological approach and analyses as the free HP system, demonstrating the effects of charge scaling implementation. RMSD analysis of the system provided a general overview of structural changes in both protein and ligand structures from the start of the simulation, RMSD was calculated for the protein backbone and ligand heavy atoms with the reference to the starting structure of the complex (Figure 5). For the simulations with TIP3P, there are high fluctuations at 7 μs with the RMSD reaching nearly 20 Å, indicating large shifts in the binding pose. The RMSD, however, did decrease afterwards, indicating the ligand to be in a binding pose similar to the starting point by the end of the simulation. This behavior is similar to the non-charge-scaled data (in orange, Figure 5). The key difference in the charge-scaled simulations is the RMSD variance is greater. All the simulations using explicit models behave in this way, with greater variability in RMSD values suggesting weaker interactions when compared to the relatively fixed binding of systems with explicit models without charge scaling. The RMSD and RMSF mean values across each simulation can be found in Tables S1 and S2.

**Figure 5:**
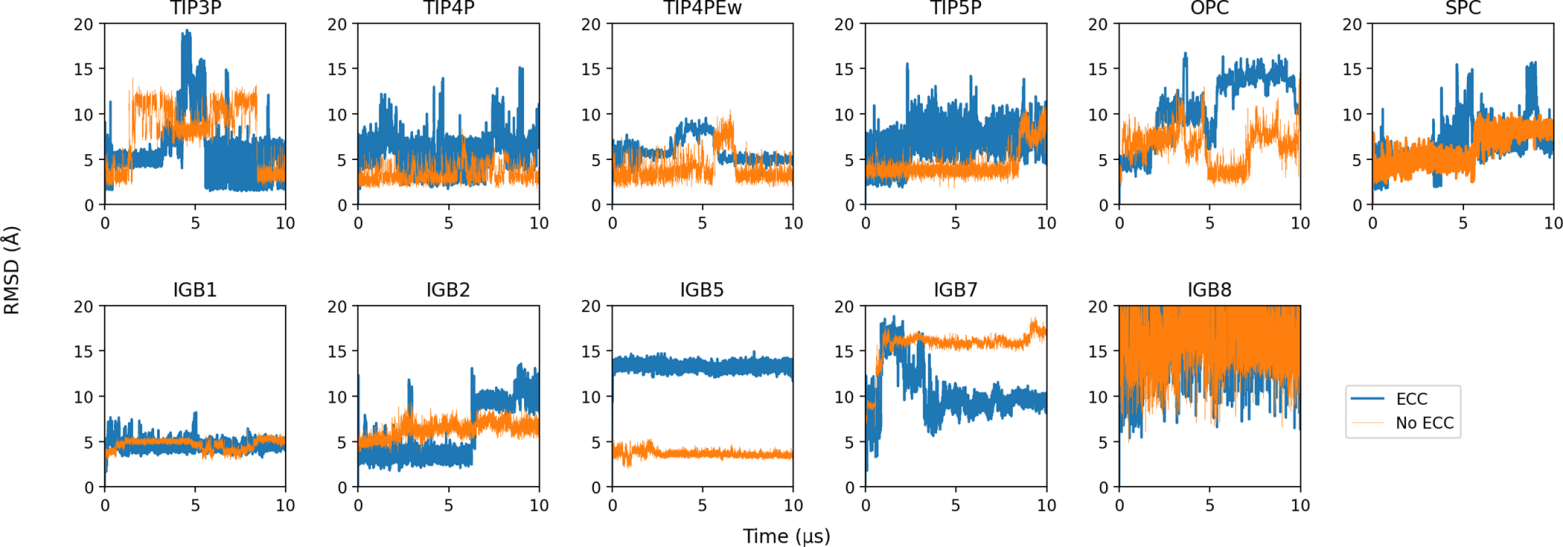
Ligand RMSD values for each simulation: the charge-scaled system in blue, and the data from prior work without charge scaling was plotted over in orange to highlight key differences.

Simulations with the implicit models IGB1 and IGB8 have similar patterns of RMSD to the non-charge-scaled simulations; however, IGB2, IGB5 and IGB7 demonstrate largely different patterns of behavior and likely occupy binding poses dissimilarly to that of the non-charge-scaled models.

Contacts analysis was also carried out, using a 7 Å distance cutoff in cpptraj, to gain greater insight into the binding under each solvent model, and the results mirrored the RMSD, with non native contacts increasing with RMSD showing how the binding pose changed over time, Figure 6. When comparing this to the non-charge-scaled work, the results are similar, once the RMSD fluctuations are taken into account. The key difference is the higher and more variable fluctuations in the non-native contacts, which would be expected given the already higher RMSD values and the weaker binding as a result of scaled down charges.

**Figure 6:**
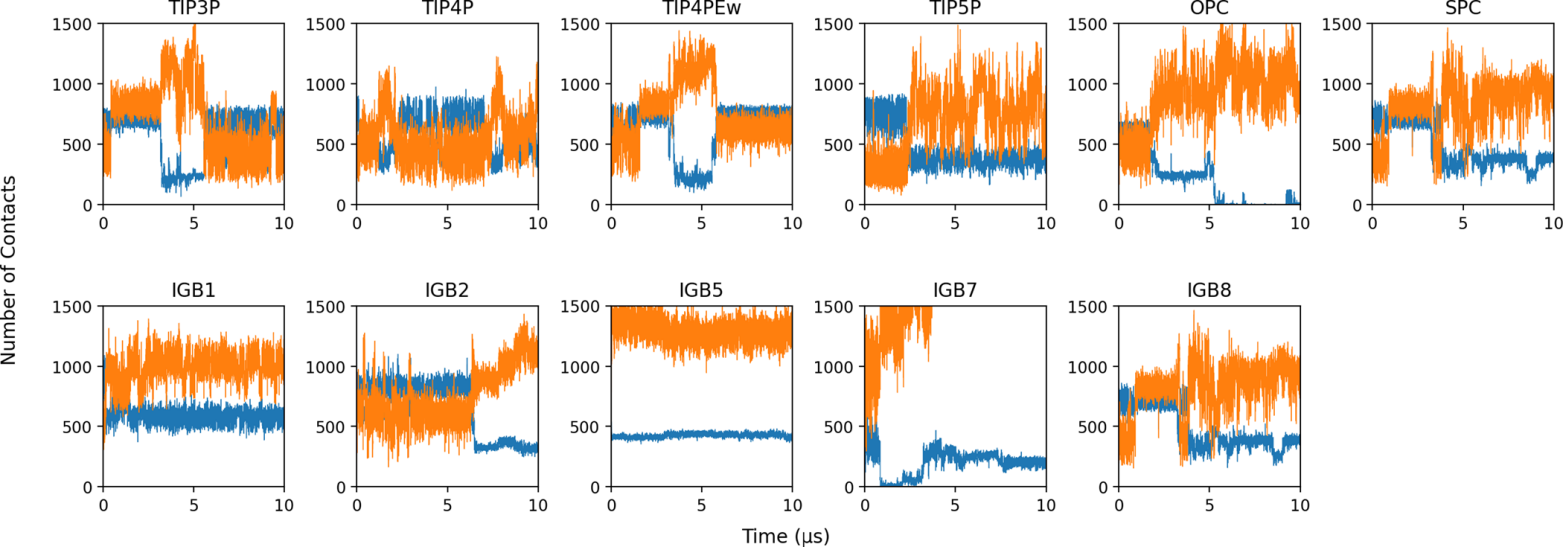
Number of protein–ligand contacts in the MD simulations with charge scaling implemented, native in blue and non-native in orange.

Hydrogen bond analysis of the systems identified a number of more specific details of how charge scaling alters the behavior of GAGs and the interactions between HP and the protein FGF (Figure 7). Both the total number of hydrogen bonds and specific hydrogen bond formation were calculated, and the total hydrogen bonding showed a substantial reduction in the charge-scaled simulations, the averages halving with most systems.

**Figure 7:**
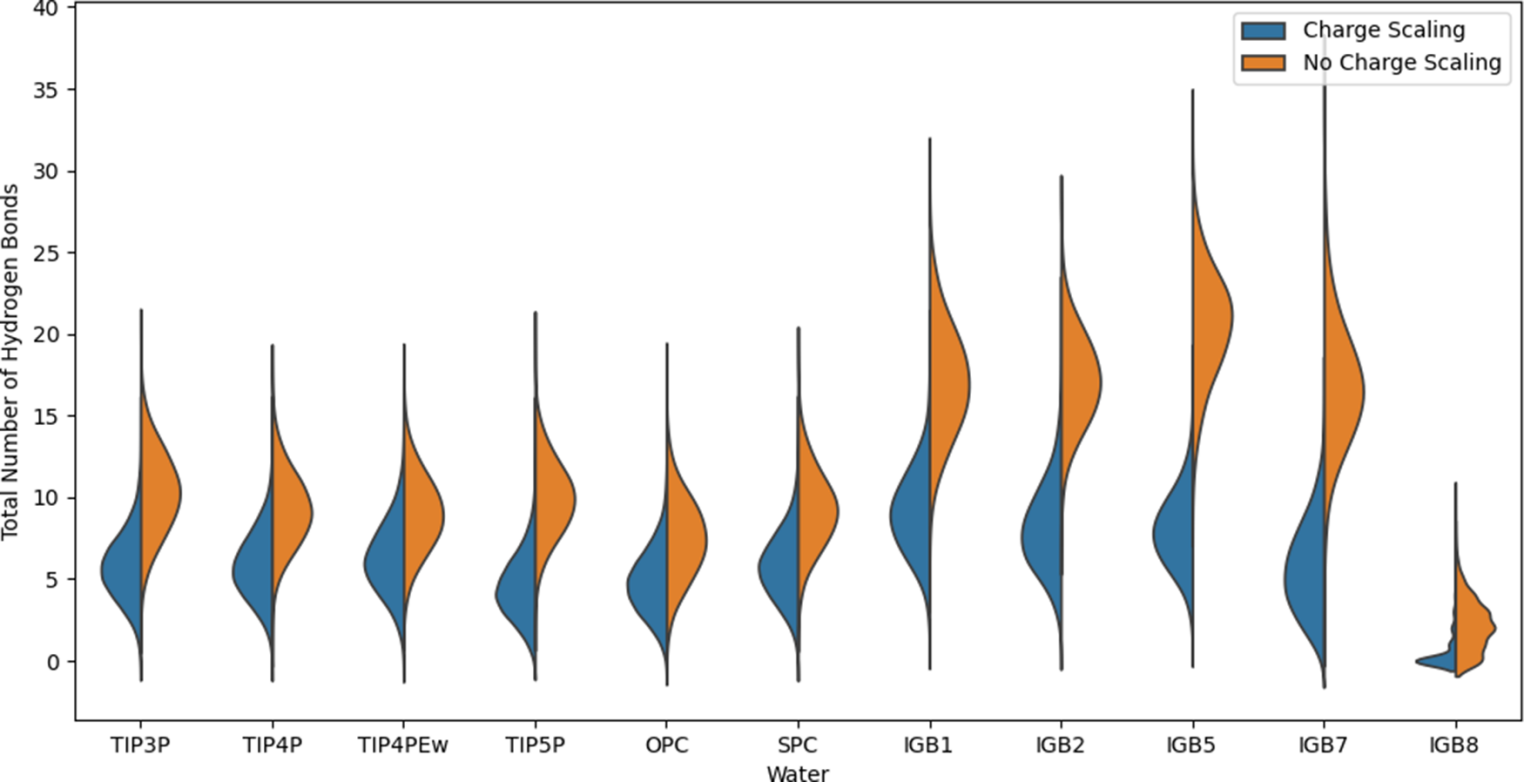
Distribution of the total number of hydrogen bonds present at any given time in a simulation represented by a violin plot. Each plot represents a different water model used in the simulation plotted. Data for the non-charge-scaled simulations plotted from Anila and Samsonov 2024.^20^

The specific hydrogen bonding pairs were also investigated and represented in heatmaps for the simulation with the TIP3P solvent (Figure 8). However, the changes are not uniformly distributed through the binding site, there are specific changes, such as the interactions between Arg121 and Arg45. To understand what these changes mean, we can see how the binding pose changes between charge-scaled and non-charge-scaled simulations to identify the mechanism that leads to the distinction in hydrogen bonding profile.

**Figure 8:**
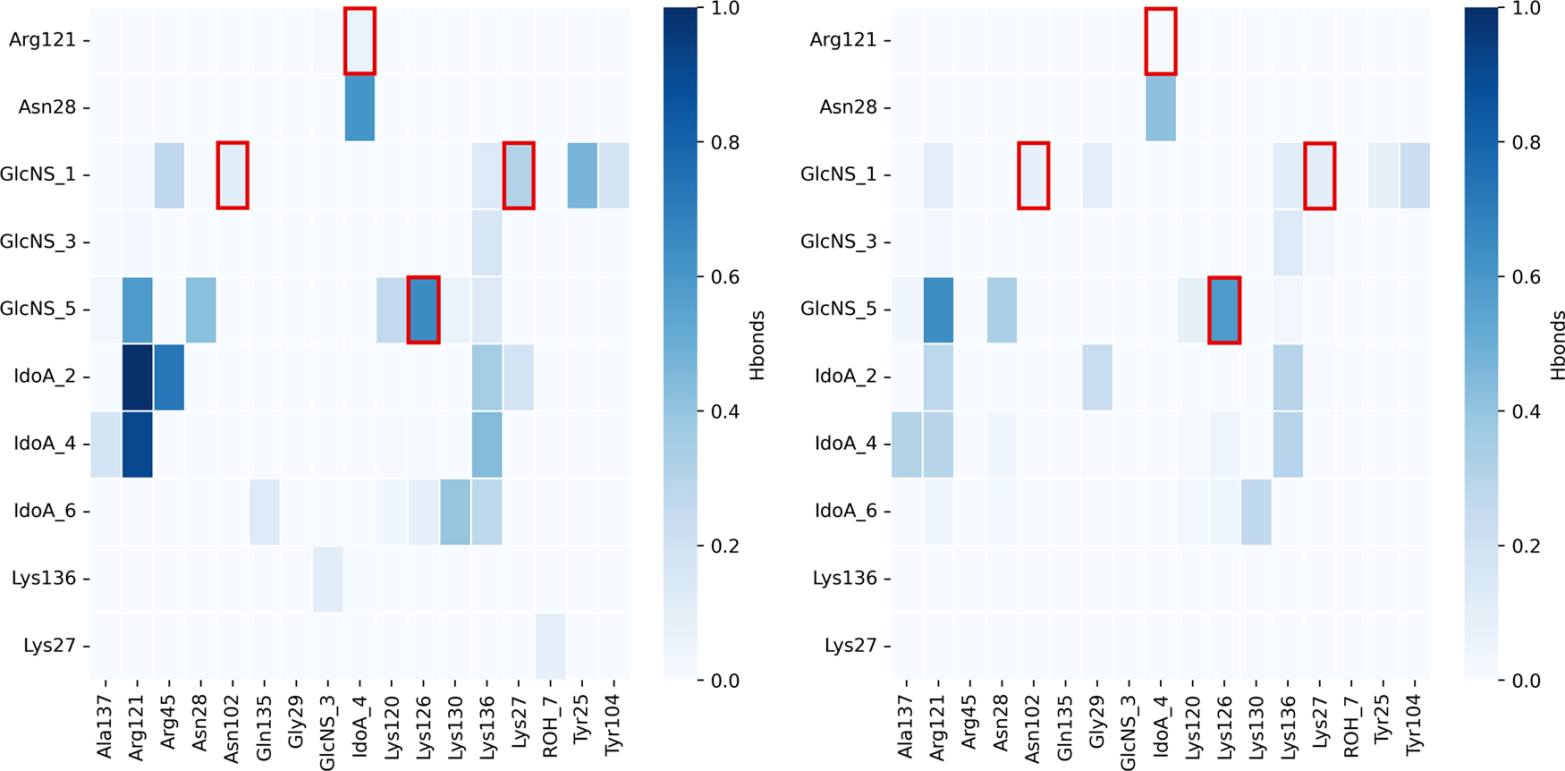
Total number of protein–GAG hydrogen bonds for each residue pair, plotted for both simulations done without charge scaling (left) and with charge scaling (right). The axes represent hydrogen bond pairs with donors on the *x*-axis and acceptors on the *y*-axis. The hydrogen bonds found in the original X-ray structure are highlighted in red for both plots.

Here, the binding poses are illustrated at various time points (Figure 9), and we can see that the HP in charge-scaled system is distant from Arg45 and without charge scaling has residues in contact with Arg45. HP both in the charge-scaled and non-charge-scaled simulations interacts with Arg121 via formation of a cage of sulfates around the positive arginine moiety; however, given the results of the contact analysis and hydrogen bonding (Figures 5 and 8), this Arg121 interaction is more stable in the non-charge-scaled system. The binding poses we see align with contact analysis in the charge-scaled system, while not binding in the same quantity as without charge scaling, it does not deviate as drastically from the starting structure to form a consistent alternative binding pose with Arg45. These results could indicate a higher degree of specificity in the system where charge scaling has been applied, while the overestimated electrostatic interactions in simulations without charge scaling lead to stronger interactions with charged residues like that seen with Arg121, Arg45, and the sulfate groups of HP. With the charge scaling implemented, the interactions are weaker but more specific to fewer binding residues on the protein (as shown by the hydrogen bond data) and, therefore, the observed conformations are less likely to be trapped within local minima.

**Figure 9:**
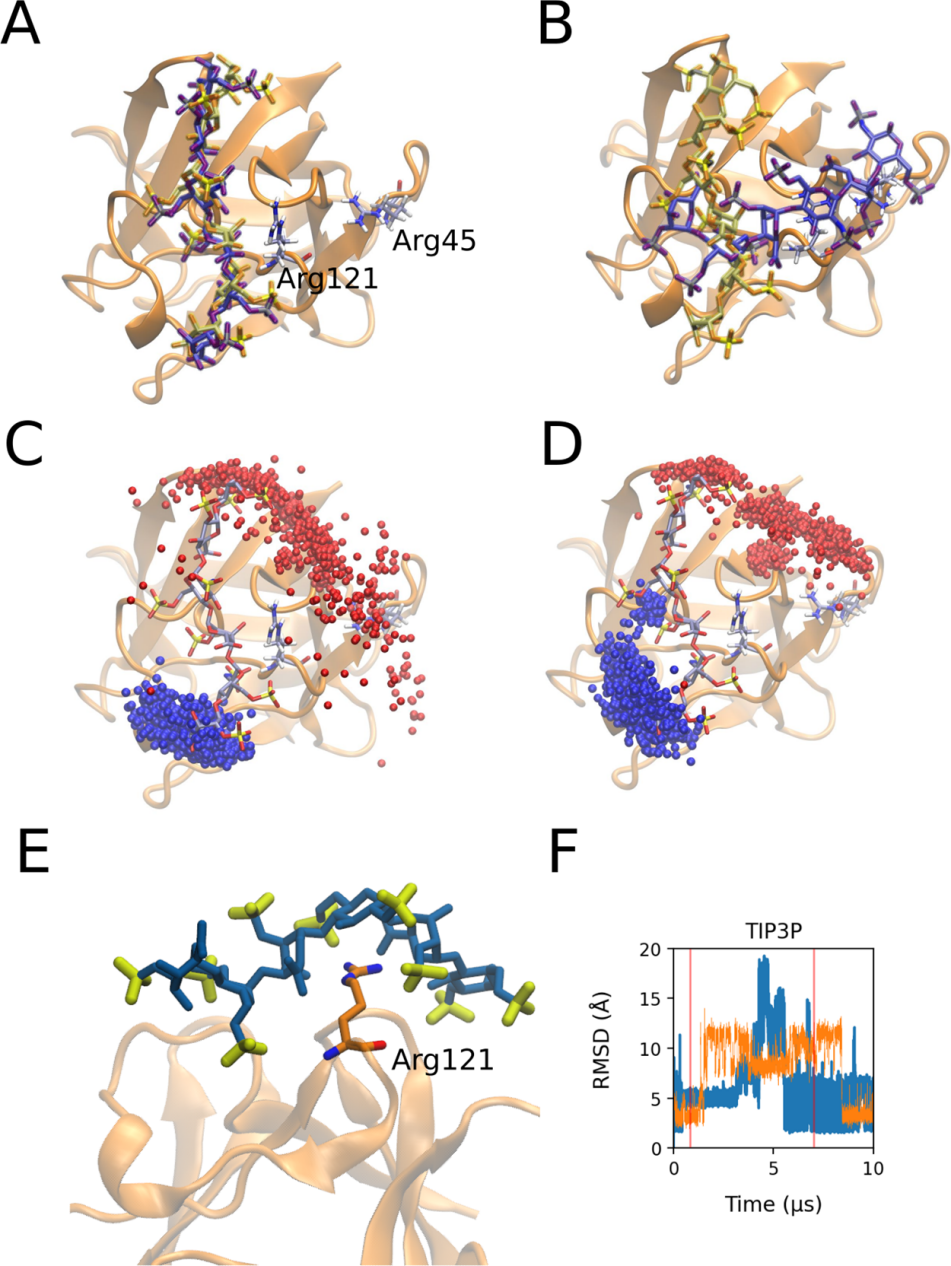
Structure of FGF and HP complex taken from the simulations with TIP3P. The HP is shown bound at 1 μs in panel (A), the charge-scaled simulation in yellow and the non-charge-scaled system in blue with data taken from Anila and Samsonov,^20^ panel (B), illustrates the difference in pose during the part of the MD simulation corresponding to high RMSD values, at 7 μs, in the data obtained with the non-charge-scaled model, where Arg121 and Arg45 form hydrogen bonds with HP. In C and D, the nonreducing end atom (in blue) and the reducing end first atom (in red) are plotted over the trajectory to highlight differences in movement, charge-scaled simulation in C and without charge scaling in panel (D). Panel (E) demonstrates the relative placement of Arg121, HP and sulfates when the non-charge-scaled HP structure is in the position shown in panel (B). The RMSD for the TIP3P system is shown in panel (F) with red bands to mark the 1 and 7 μs interval snapshots were taken from. The blue corresponds to the charge-scaled system and orange corresponds to the non-charge-scaled data.

A charge distribution analysis was also carried out to understand how the charge scaling impacted the distribution of counterions in solution and how the ion-paring behavior was changed. Many more of the ions are densely distributed around the ligand with no charge scaling (Figure 10). MM/GBSA was also carried out, and the ΔG values have been reported in Table S5 and show how the charge scaling leads to a weaker binding energy close to the relative scaling applied in the ECC method.

**Figure 10:**
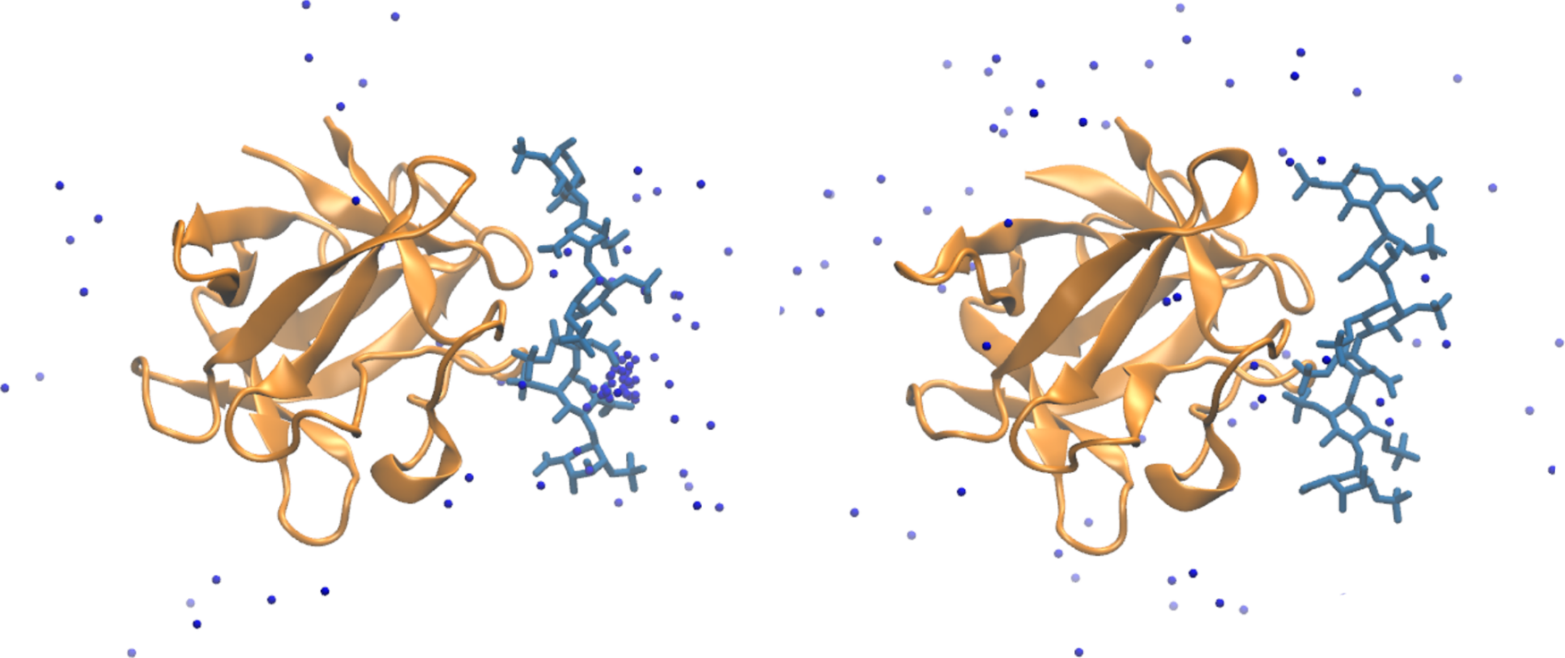
Representative ensembles of the FGF-HP complexes for both the non-charge-scaled system on the left and the charge-scaled system on the right, the snapshot of the protein and HP are shown for understanding the relative position of ions in blue. The ions shown are a sample of 100 frames of the production simulation to provide insight into their distribution.

### Heparin with Calcium Ions

To probe how charge scaling may alter the behavior of HP in the presence of calcium ions, a similar set of analyses were carried out on simulations with charge-scaled HP using the same series of solvent models. It has also been demonstrated that calcium ion presence contributes to the folding of the structure,^21^ an issue explored here in the context of charge scaling. In these simulations, the results were similar, but the differences between the charge-scaled and non-charge-scaled systems were exacerbated. The EED was found to be higher with charge scaling in this system (Figure 11). The mean values for the general descriptors can be found in Tables S3 and S4 for comparison between charge-scaled and non-charge-scaled data. A potential explanation of the shift in behavior is likely a result of the calcium in solution mediating intramolecular interactions and, therefore, being sensitive to the changes in charges that dictate electrostatic interactions. In the charge-scaled system, calcium acts as an electrostatic buffer, reducing the impact of any intramolecular interactions and leading to a more extended structure. In the case of the system without charge scaling, however, the stronger electrostatic interactions are capable of capturing calcium ions within the folded structure. Captured calcium ions function as salt bridges, stabilizing the conformation in a compressed state and holding distant HP residues together; with charge scaling implemented, these interactions are not stable, and therefore, a more extended conformation is more energetically favorable. The extended lengths observed with the charge-scaled systems align with experimental findings^22,35,42^ in a similar way to the HP simulated without calcium ions, discussed earlier. The performance of the charge scaling scheme is still superior to the one of the nonscaled scheme even when external ionic charges in the form of the calcium ions are introduced to a system.

**Figure 11:**
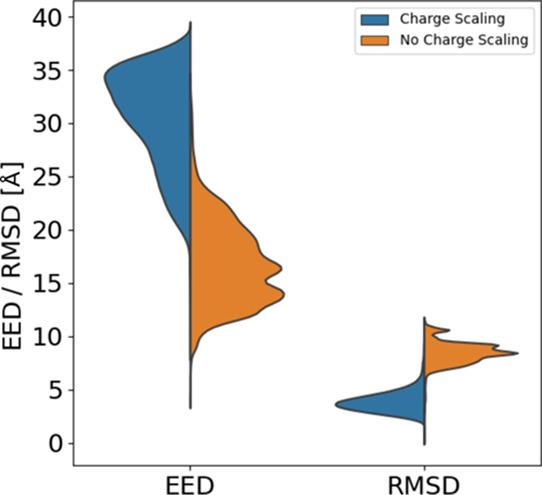
EEDs and RMSD values of the HP system with the TIP3P water model simulated with calcium ions.

## Conclusions

Overall, the results presented here cover three scenarios in which charge scaling has been implemented, and the effects were quantified. The behavior of HP when free in solution, when bound to a protein, and in the presence of calcium ions changes when the ECC protocol is applied, and these effects have been assessed within the context of solvent model choice to provide a more complete understanding of how GAG simulations can be optimized. In general, the ECC protocol brings descriptors of HP closer to the experimental values while also reducing the strength of intra- and intermolecular interactions.

The simulations of free HP provide the strongest evidence for the benefits of utilizing charge scaling, shifting EED values significantly closer to the experimental ones, and eliminating the artifactual folding behavior seen in the same simulations of HP without charge scaling. This result is of particular importance for optimization of GAG simulations due to the widespread use of the TIP3P model, which no longer exhibits this improper folding with ECC implemented. However, the charge scaling approach did not completely remedy the over extension of the HP molecules observed in the simulations with implicit models, demonstrating that a more detailed explicit solvent model is still required to accurately simulate GAGs. It is also important to consider that the artifactual behavior manifested with the EED parameter is due to the combination of the TIP3P solvent and the unscaled charges set. Previous work without charge scaling cited OPC and TIP5P as optimal model choices due to the lack of HP unnatural folding. The use of more computationally demanding models like TIP5P, however, can be avoided as the data here show how TIP5P-like GAG behavior can be gained with simpler models by implementing charge scaling.

Investigation of the protein–GAG system provided details of how charge scaling alters the interface and interactions involved in protein-GAG binding. The contact and RMSD analyses demonstrated how stability differed across water model choice, generally showing a reduction in stability with charge scaling, as would be expected with the decreased absolute charge values. However, the reduced hydrogen bonding and specific pattern of hydrogen bonds provided insights into the potential benefits of charge scaling. Within the TIP3P system, the charge-scaled simulation demonstrated more specific binding in which the binding pose remained closer to the starting structure. However, this does not clearly suggest if the limitations of the use of the TIP3P water model could be solely addressed by the application of the charge scaling scheme. The statement about the specificity should be, therefore, taken carefully in the context of the particular molecular system studied with a specific set of the parameters analyzed. Finding a reduced total number of hydrogen bonds suggests weaker binding; however, there was also a reduced variety in the types of hydrogen bonding pairs formed. The identification of specific hydrogen bonds disappearing with charge scaling, along with a more stable binding pose demonstrated by the RMSD, suggests that the use of ECC leads to more specific binding and better reproduction of the crystal structure-bound pose.

In the third scenario explored, HP simulated with calcium ions addressed how the changes in the ECC protocol impacted GAG–calcium ion interactions. The exacerbated folding in simulations using models like TIP3P presents a trial for simulating GAGs, due to the importance of calcium ions and the need to capture GAG behavior in the presence of these ions. The results demonstrate how ECC applied to HP led to improvements qualitatively similar to HP without calcium, while under the same conditions without the charge scaling, the structure was more improperly folded.

To verify the results, a sample repeat was carried out, simulating the free HP system with TIP3P for both the charge-scaled and -unscaled systems, for 1 μs. The general descriptors were found to align with the original 10 μs results with the exception to the EEDs (Table S6). The EEDs differ due to the length of the simulation, as reported in prior work,^19^ the collapse of HP requires longer simulation times. However, the repeated hydrogen bond analysis and other descriptors in Table S6 and Figure S5 effectively demonstrate the reproducibility of these results with longer MD simulations. Potential issues regarding counterion underscreening were also considered, as previous literature has explored this in the context of charge scaling, showing the ECC protocol to be sensitive to the underlying system and screening effects.^43^ The ionic strength of the systems used here (168 mM) was close to the physiological standard of 150 mM. Interpretation of the data also requires understanding the fact that the charge scaling affects intramolecular interactions between the atoms of HP. In particular, charge scaling decreases the repulsion of the charged groups within the molecule, underlining the significance of the observed effect of the charge scaling on the interaction with solvent and counterions, which in total leads to the amelioration of the artifactual HP conformation behavior (collapse).

In conclusion, the data presented here provide a comprehensive overview of how the utilization of charge scaling can be used to improve the accuracy of GAG simulations without compromising on computational resource demands, with the specific artifactual behavior being reported in previous MD simulations^19,21^ being ameliorated. In all three sets of simulations, the use of the charge scaling yielded HP behavior closer to the experimental values and allowed simpler solvent models like TIP3P to perform at a level similar to that of more complex and demanding models like TIP5P. While other GAGs that are less sulfated than HP may not be as sensitive to the charge scaling effects demonstrated here, the common GAG properties make it likely that any differences will be quantitative, and the qualitative improvement would likely be retained. This information can be used in future work to improve the simulation design and highlights the importance of quality solvent models and the accurate capture of electrostatic interactions when simulating GAGs.

## Supplementary Material



## Data Availability

The data supporting this article were generated using AMBER pmemd and analysis was carried out with the AMBER suite and AmberTools.^41^ Figures were prepared with Inkscape and visualization was carried out with VMD. The inputs and files needed to reproduce this work are available in the manuscript and supporting files. All the software other than the AMBER suite is free of charge. AMBER software can be obtained from http://ambermd.org/GetAmber.php. Inkscape can be downloaded from https://inkscape.org/. VMD can be downloaded from http://www.ks.uiuc.edu/Research/vmd/.

## Notes

Plots of the EED, RMSD, and total hydrogen bonds over time and dihedral angles data for the HP system and corresponding descriptor values for the FGF-HP and HP calcium systems (PDF)

## Abbreviations

ECC: electronic continuum correction
EED: end-to-end distance
FGF: fibroblast growth factor
GAG: glycosaminoglycan
HP: heparin
MD: molecular dynamics
NPT: isothermal isobaric ensemble
PG: proteoglycan
PDB: Protein Data Bank
RMSD: root mean square deviation
