# Supplementary material for: Solvent Models and Charge Scaling: Benchmarks for Molecular Dynamics of Glycosaminoglycans
Source: J Phys Chem B. 2026 Apr 29;130(19):5002–12. doi: 10.1021/acs.jpcb.6c01164 (PMC13309006; doi:10.1021/acs.jpcb.6c01164)
Supplement: Supplementary file 1 [file jp6c01164_si_001.pdf]

Solvent Models and Charge Scaling: Benchmarks for Molecular Dynamics of Glycosaminoglycans: Supplementary Material

Jacob A. Clark[1] and Sergey A. Samsonov*[1]

[1]Faculty of Chemistry, University of Gdansk, Wita Stwosza 63, Gdansk, 80-308, Poland

*Email: sergey.samsonov@ug.edu.pl

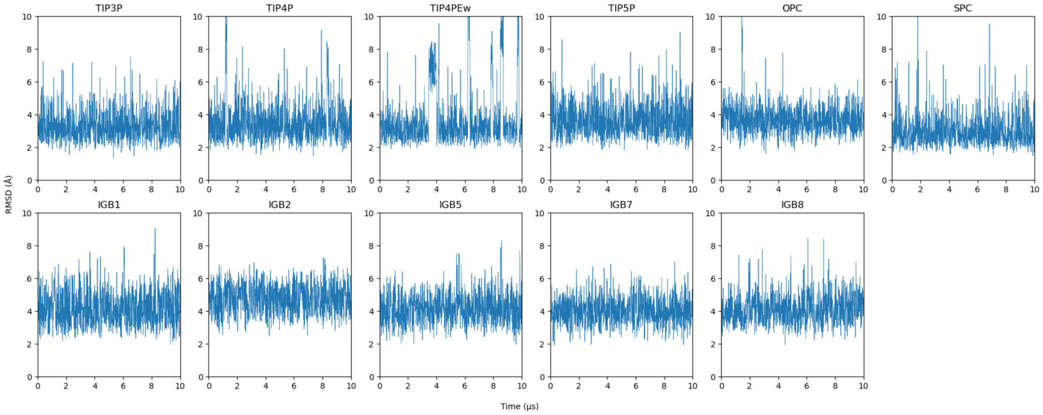

Figure S1: RMSD for each simulation of unbound heparin with line plots labelled with the respective solvent model used.

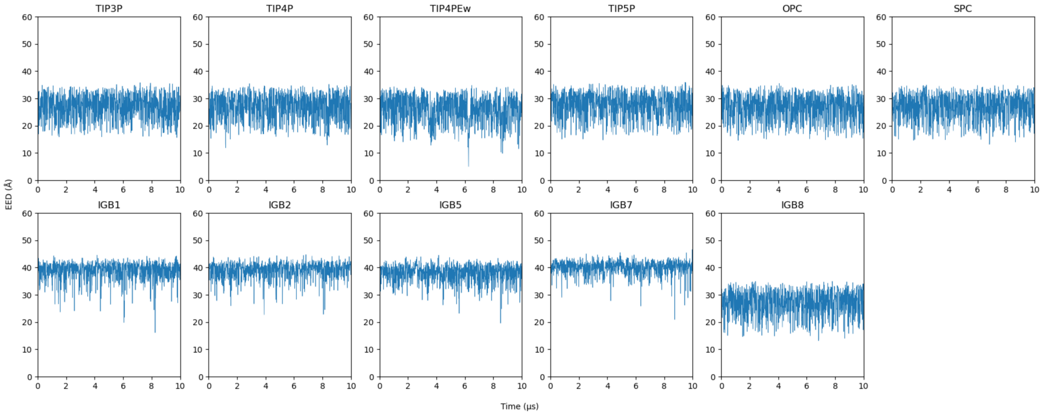

Figure S2: EED for each simulation of unbound heparin with line plots labelled with the respective solvent model used.

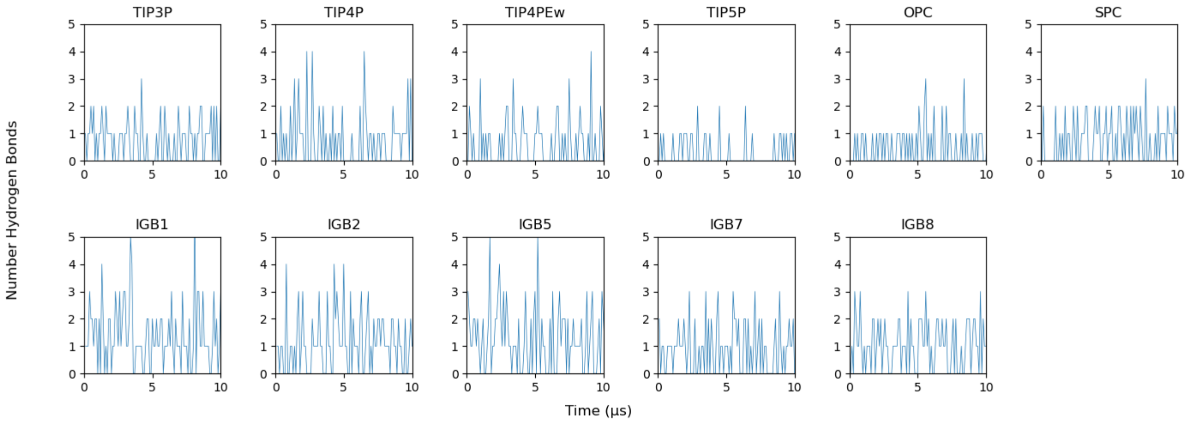

Figure S3: Hydrogen bonds over time, represented in line plots for each simulation and the respective solvent models used labelled.

Table S1: Comparison of general descriptors demonstrating protein stability in simulations using explicit solvent models in the FGF-HP system

	TIP3P	TIP4P	TIP4P-Ew	TIP5P	OPC	SPC/E
RMSD (Å)	3.0±0.6	3.6±0.7	3.2±0.6	3.1±0.4	2.7±0.8	3.1±1.0
Fluct (Å)	1.0±0.4	1.1±0.5	1.0±0.5	1.0±0.4	1.0±0.5	1.1±0.5

Table S2: Comparison of general descriptors demonstrating protein stability in simulations using implicit solvent models, in the FGF-HP system.

	IGB1	IGB2	IGB5	IGB7	IGB8
RMSD (Å)	3.2±0.4	3.7±1.4	4.2±0.5	2.4±0.6	3.2±0.7
Fluct (Å)	1.3±0.5	1.9±0.7	1.6±0.7	4.4±1.9	2.7±1.1

Table S3: Comparison of general descriptors in simulations using explicit solvent models in the HP-Ca system

	TIP3P	TIP4P	TIP4P-Ew	TIP5P	OPC	SPC
RMSD (Å)	8.4±1.1	7.5±1.0	7.4±1.0	7.8±1.4	8.9±0.5	8.4±2.1
Dist (Å)	16.8±3.9	17.0±7.1	16.9±7.5	19.4±4.7	21.3±2.2	22.9±4.1
Fluct (Å)	4.0±0.8	4.4±1.8	4.0±1.4	4.6±1.3	2.9±0.5	4.8±0.8

Table S4: Comparison of general descriptors in simulations using explicit solvent models with charge scaling in the HP-Ca system.

	TIP3P-ECC	TIP4P-ECC	TIP4P-Ew-ECC	TIP5P-ECC	OPC-ECC	SPC-ECC
RMSD (Å)	3.9±1.0	6.0±3.3	6.6±3.3	3.5±1.1	3.3±1.3	3.7±1.2
Dist (Å)	30.4±2.2	26.5±6.7	23.6±6.6	32.2±2.2	31.0±2.3	33.0±2.6
Fluct (Å)	3.2±0.8	5.7±1.5	5.6±1.5	3.2±0.8	3.0±0.7	3.1±0.8

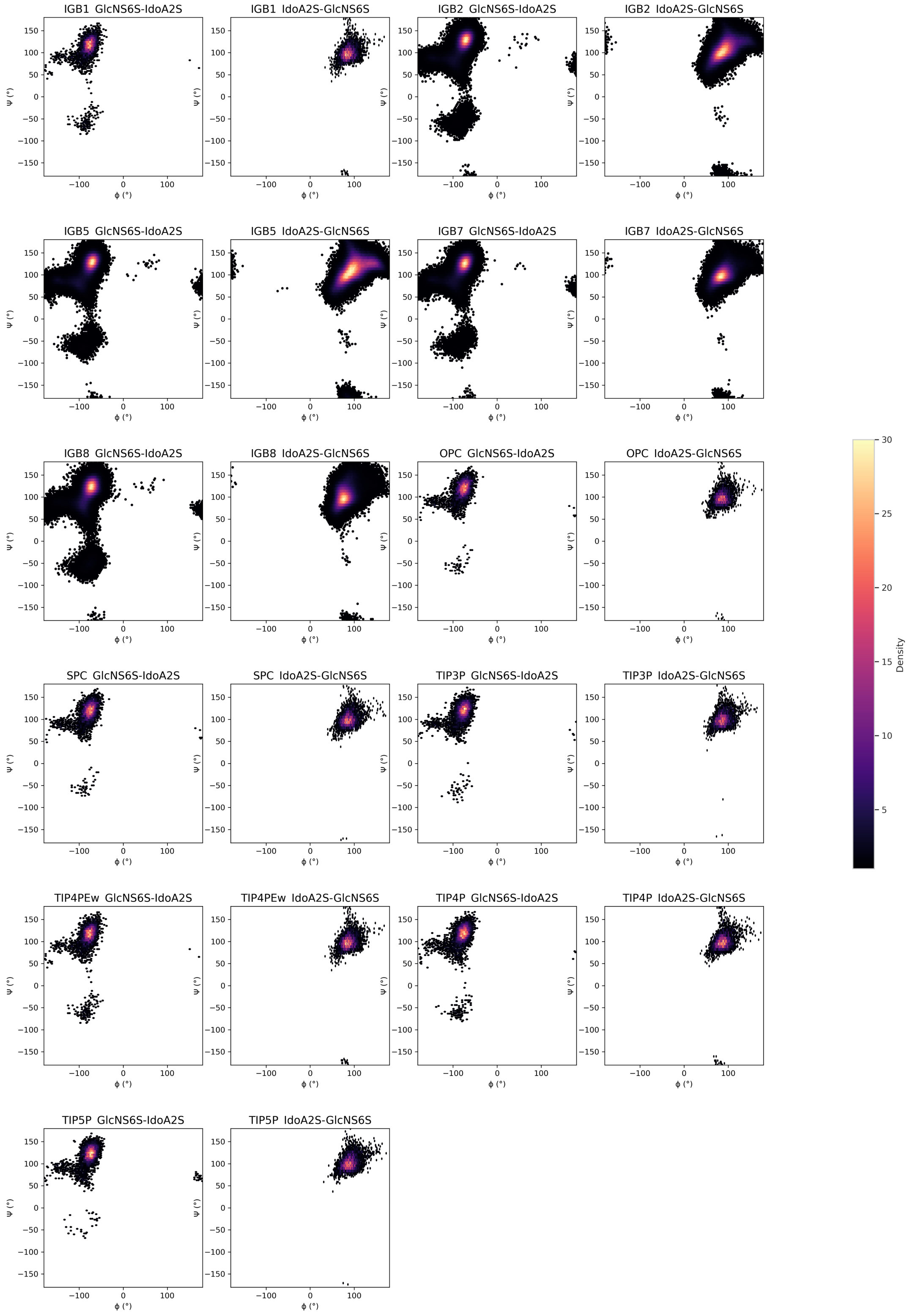

Figure S4: Glycosidic linkage dihedrals for the Heparin simulation using the solvent model TIP3P.

Table S5: MM/GBSA calculated ΔG for the charge scaled and non-charge systems.

	Charge Scaled	No Charge Scaling
	Energy (kcal/mol)	Energy (kcal/mol)
TIP3P	-85±13	-107±13
TIP4P	-79±10	-111±11
TIP4PEw	-91±10	-107±13
TIP5P	-77±14	-107±12
OPC	-75±10	-92±15
SPC	-94±15	-116±14
IGB1	-125±9	-170±15
IGB2	-117±17	-212±14
IGB5	-164±15	-261±22
IGB7	-103±28	-195±33
IGB8	-13±8	-33±9

Table S6: Comparison of general descriptors in the replica simulations with and without charge scaling simulations

	With Charge Scaling	No Charge Scaling
RMSD (Å)	3.8±1.1	6.5±2.3
Fluct (Å)	3.1±0.8	5.4±1.4
Radgyr (Å)	12.7±0.7	11.0±1.4
EED (Å)	27.0±4.6	25.6±5.2

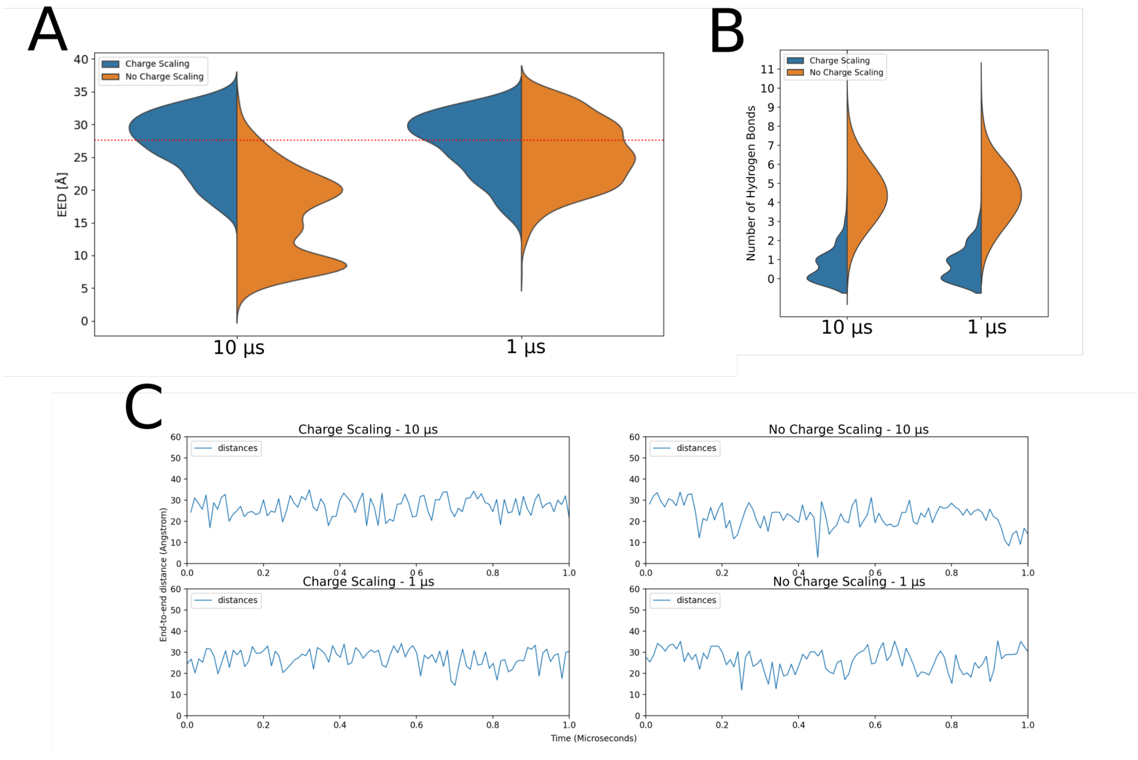

Figure S5: Comparison of the general descriptors between the original 10 μs run and the 1 μs replica, the EED shown in A and the Number of Hydrogen bonds in B. A time series of the EEDs is shown in C.
